# Dataset of soil bacterial compositions and biochemical properties of a Moso bamboo forest under mulching-intensive management

**DOI:** 10.1016/j.dib.2019.104973

**Published:** 2019-12-11

**Authors:** Weicheng Li, Xinli Tian, Haiyan Sheng, Desy Ekawati, Yan Zhou, Rui Zhang

**Affiliations:** a China National Bamboo Research Center, Key Laboratory of High Efficient Processing of Bamboo of Zhejiang Province, Hangzhou 310012, China; b College of Life Sciences, Southwest Forestry University, Kunming 650224, China; c Hangzhou Environmental Protection Science Institute, Hangzhou 310005, China; d Center for Social Economic Policy and Climate Change Research and Development, Forestry and Environment Research Development and Innovation Agency, Bogor 16610, Indonesia

**Keywords:** Microbe, Soil biochemical properties, Microbial biomass stoichiometry, *Phyllostachys edulis*

## Abstract

Bacterial 16S rRNA dataset of Moso bamboo forest was formed by 20 soil samples in four management modes corresponding to the soil properties data of each soil sample such as concentrations of soil organic carbon (SOC), total nitrogen (TN), total phosphorus (TP), available phosphorus (AP), NH_4_^+^-N, NO_3_^−^-N, water-soluble organic carbon (WSOC), Water-soluble organic nitrogen (WSON), microbial biomass carbon (MBC) and microbial biomass nitrogen (MBN), and soil water content (SWC) and pH value. Due to the special climate in the northern edge of subtropical zone and the characteristics of non wood and non grass of bamboo plants, our data set is helpful for the further studies of soil management, microhabitats variations responding to global carbon, nitrogen and phosphorus cycle. The data is related to the research article “*Response of bacterial compositions to soil biochemical properties under mulching-intensive management in a Phyllostachys edulis forest*” [1].

**Table:** Specifications Table

Subject	Environmental Science (General)
Specific subject area	Soil microbial ecology and forest management
Type of data	Table
How data were acquired	1. DNA was extracted using HiPure soil DNA kit B. 2. KAPA HiFi HotStart ReadyMix was used in The polymerase chain reaction (PCR). 3. Library construction and Illumina HiSeq 2500 sequencing were setup. 4. The obtained DNA sequence reads were trimmed using PANDAseq. 5. The sample sequences were combined using QIIME 1.9.1 software.
Data format	Filtered
Parameters for data collection	The hypervariable V3/V4 regions of the microbial 16S rRNA gene were amplified using the forward primer 5′-CCTACGGGNGGCWGCAG-3′ and the reverse primer 5′-GACTACHVGGGTATCTAATCC-3′ [2].
Description of data collection	Five 10 m × 10 m replicate plots were set up in each of the four management modes, and a total of 20 plots were established. Soil samples were collected from a depth of 0–20 cm. Nine randomly selected points in each plot were sampled, and the samples were thoroughly mixed to combine one replicate. A portion of the sieved sample was stored at −78 °C in an ultra-low temperature freezer for DNA extraction and high-throughput sequencing; another portion of the sieved sample was air-dried. The remaining portion was stored at 4 °C and used for determining the microbiological parameters.
Data source location	City/Town/Region: Xiaopu in Changxing County of Zhejiang Province Country: China Latitude and longitude (and GPS coordinates) for collected samples/data: 30°58′N, 119°45′E
Data accessibility	With the article Direct URL to experimental data: https://data.mendeley.com/drafts/
Related research article	Author: Weicheng LI, Xinli TIAN, Haiyan SHENG, Desy Ekawati, Yan Zhou, Rui Zhang Title: Response of Bacterial Compositions to Soil Biochemical Properties under Mulching-Intensive Management in a *Phyllostachys edulis* Forest Journal: Applied Soil Ecology DOI

**Table:** 

**Value of the Data** • The dataset provides the variations of bacterial compositions both on phylum and genus levels among four management modes. Meanwhile, it also showed the changes of soil biochemical properties corresponding to bacterial communities. Further, it suggests that the best management mode is the combination of mulching and alternation. • Researchers and policy makers related to soil fertility, soil microorganism, environmental pollution and forest management can all get benefits from it. • Firstly, the community analysis of Archaea and Fungi needs to be added to further studies on the impact of mulching. Secondly, Isolation and identification of microbe that decompose organic matter in the mulching bamboo forest is potential. Thirdly, the relations between different fertilization and the output of bamboo forest can find the optimum fertilization amount and structure of bamboo forest. • Those data can be a part of a meta-analysis of soil microbe or soil elemental cycle.

## 1 Data

The dataset contains information on soil biochemical properties (supplementary materials M1), Phylum distribution of dominant soil bacteria (OTU≥1.0%) in the four management modes (supplementary materials M2), 16S rRNA profiles of 27 phyla (supplementary materials M3) and 197 genera (supplementary materials M4) selected from 20 sample plots in four management modes.

## 2 Experimental design, materials, and methods

### 2.1 Experimental design

The mulching areas were operated annually in the following way. The density of 1- to 5-year-old mother individuals was approximately 2700–3300 per ha in the mulching area. In early April, 1500 kg of quicklime was sprayed over each ha. Compound fertilizer (750 kg per ha, N:P_2_O_5_:K_2_O = 15:15:15) was applied to assist rooting from May to June. Simultaneously, shallow ploughing was carried out in the 10–15 cm soil layer. A total of 1500 kg per ha of bamboo shoot compound fertilizer was applied from August to September (the same component mentioned above). From October to November, flood irrigation (300–600 t per ha) saturated the soil of the bamboo forest. Then, 7000 kg per ha organic chicken manure fertilizer was evenly spread. The dry matter of chicken manure contained 19.3% crude protein, 2.4% fat, 13.1% ash, 10.3% carbohydrate, 7.2% fibre, 2.45% nitrogen (N), 2.66% phosphorus (P) and 0.91% potassium (K). At the same time, 1500 kg per ha of bamboo charcoal fertilizer (N, 11.9 wt.; P_2_O_5_, 4.8 wt.; K_2_O, 5.7 wt.; pH, 8.13–8.50; conductivity, 5.47 mS cm^−1^; moisture content, 30.5%; and C/N, 14.7) was dispersed. From November to December, the mulching area was mulched with double layers. Rice straw was placed in the lower 15–20 cm layer, and water was applied to increase the temperature (15–20 °C). Rice husks were placed in the upper 20–25 cm layer, which served as a heat preservation measure. At the end of April the following year, the two organic mulch types were removed immediately after the bamboo shoots were excavated. Other residual materials were dug into the soil for fertilization [1].

The density of 1- to 5-year-old mother individuals was approximately 2200–3000 per ha in the alternation area. The proportion of bamboo plants in the 2-, 3- and 4-year-old age classes reached 80%. From May to early June, 750 kg per ha of compound fertilizer was applied for rooting (the same component). Bamboo shoot fertilizer was applied from August to September, 750 kg per ha of compound fertilizer was applied (the same component), and the area was smoothed to remove old roots and stumps.

Sampling plots were established with four management modes, each of which was 0.33 ha. The four modes included the following: CK (a control area, 50 m from the mulching-alternation area, no fertilization and mulching, only the random digging of bamboo shoots in the past 12 years), MU (mulching mode, the second year of mulching after 2 years of alternation), AL (alternation mode, the first year of alternation after 3 years of mulching), and LT (long-term mulching mode, 2011–2017, fertilization and the other operations were the same as those in the mulching area).

Soil samples were taken in April after removing the organic mulch. Five 10 m × 10 m replicate plots were set up in each of the four management modes, and a total of 20 plots were established. An interval of 10–20 m between each plot served as a buffer. Soil samples were collected from a depth of 0–20 cm by boring with an auger. Nine randomly selected points in each plot were sampled, and after surface litter and impurities were removed, the samples were thoroughly mixed to combine one replicate. The soil samples were stored at 4 °C in a sample box and transported to the laboratory within 6 h of collection. The samples were sieved (2-mm sieve) to homogenize the samples and remove visible roots. A portion of the sieved sample was stored at −78 °C in an ultra-low temperature freezer until further use for DNA extraction and high-throughput sequencing; another portion of the sieved sample was air-dried. The remaining portion was stored in a plastic bag at 4 °C and used for determining the microbiological parameters.

### 2.2 Soil properties

The pH was determined in a 1:2.5 (w/v) soil:water extract using a glass LE410 pH electrode (FiveEasy™ pH metre; Mettler-Toledo AG, Switzerland). The soil organic carbon (SOC) and total N (TN) concentrations were measured using a total organic carbon (TOC) analyser (vario MAX cube; Elementar Analysensysteme GmbH, Germany). Twenty grams of fresh soil subsamples were weighed, mixed with distilled water at a ratio of 2:1 (w/v), shaken for 0.5 h at 25 °C, centrifuged for 10 min at 8000 rpm, and then filtered through a 0.45 μm membrane. The filtrate was divided into three parts that were directly used to determine the following: 1) the content of water-soluble organic carbon (WSOC) using a TOC analyser (TOC-VCPH, Shimadzu, Japan); 2) the total water-soluble nitrogen (WSN) content using the same TOC analyser; and 3) the water-insoluble inorganic nitrogen (WIN; NH_4_^+^, NO_3_^−^, and NO_2_^−^) content using ion chromatography. Water-soluble organic N (WSON) was calculated as the difference between WSN and WIN. The microbial biomass carbon (MBC) and microbial biomass nitrogen (MBN) concentrations were determined using a fumigation-extraction method and calculated using a conversion factor of 2.22 [3].

### 2.3 DNA extraction and Illumina HiSeq sequencing

DNA was extracted using HiPure soil DNA kit B. The hypervariable V3/V4 regions of the microbial 16S rRNA gene were amplified using the forward primer 5′-CCTACGGGNGGCWGCAG-3′ and the reverse primer 5′-GACTACHVGGGTATCTAATCC-3′ [2]. Library construction and Illumina HiSeq 2500 sequencing were completed by Shanghai Xiangyin Biological Technology Co., Ltd. (see accompanying article for details).

### 2.4 Synthetic data

The results showed that the diversity of soil bacteria included 26 phyla and 197 genera in the four different management modes of Moso bamboo forest, among which Acidobacteria were dominant, followed by Proteobacteria. Chloroflexi, Verrucomicrobia, AD3, Actinomycetes and TM7 also had certain advantages [1]. Due to the application of organic fertilizer and compound fertilizer, the soil nutrients, such as SOC, WSOC and TN, increased in the MU, AL and LT modes. PCoA analysis showed that mulching-intensive management had a great impact on the diversity and structural composition of the soil bacteria. Mulching operations increased the number, diversity and abundance of bacteria. The Chao-1 index of the LT mode indicated that long-term mulching had an inhibitory effect on the soil bacterial community, and the excessive interference intensity exceeded the microbial recovery ability [4], which was not conducive to long-term sustainable management. Results showed that the soil bacteria had a positive gradient response to pH values, which suggested that soil pH value is one of the key factors affecting the composition and distribution of soil microorganisms [5]. Most nutrient indicators decreased under the LT mode, e.g., NH_4_^+^-N decreased significantly, while NO_3_^−^-N increased, inhibiting microbial flora activity suggesting that the application of a certain amount of N fertilizer could increase the number and activity of bacteria in soil, and excessive N fertilizer may inhibit the growth and activity of bacteria because of the imbalance of N forms.
